# Deciphering the Liaison Between Fine Particulate Matter Pollution, Oxidative Stress, and Prostate Cancer: Where Are We Now?

**DOI:** 10.3390/antiox13121505

**Published:** 2024-12-10

**Authors:** Chiang-Wen Lee, Yao-Chang Chiang, Thi Thuy Tien Vo, Zih-Chan Lin, Miao-Ching Chi, Mei-Ling Fang, Kuo-Ti Peng, Ming-Horng Tsai, I-Ta Lee

**Affiliations:** 1 Department of Respiratory Care, Chang Gung University of Science and Technology, Chiayi 613, Taiwan; cwlee@gw.cgust.edu.tw (C.-W.L.); mcchi@mail.cgust.edu.tw (M.-C.C.); 2 Chronic Diseases and Health Promotion Research Center, Chang Gung University of Science and Technology, Chiayi 613, Taiwan; ycchiang01@mail.cgust.edu.tw (Y.-C.C.); zclin@mail.cgust.edu.tw (Z.-C.L.); 3 Department of Orthopedic Surgery, Chang Gung Memorial Hospital, Chiayi 613, Taiwan; mr3497@cgmh.org.tw; 4 Department of Safety Health and Environmental Engineering, Ming Chi University of Technology, New Taipei City 243, Taiwan; 5 Department of Nursing, Division of Basic Medical Sciences, Chang Gung University of Science and Technology, Chiayi 613, Taiwan; 6 Faculty of Dentistry, Nguyen Tat Thanh University, Ho Chi Minh 70000, Vietnam; vtttien@ntt.edu.vn; 7 Division of Pulmonary and Critical Care Medicine, Chiayi Chang Gung Memorial Hospital, Chiayi 613, Taiwan; 8 Center for Environmental Toxin and Emerging-Contaminant Research, Cheng Shiu University, Kaohsiung 833, Taiwan; k6764@gcloud.csu.edu.tw; 9 Super Micro Research and Technology Center, Cheng Shiu University, Kaohsiung 833, Taiwan; 10 College of Medicine, Chang Gung University, Taoyuan 333, Taiwan; 11 Division of Neonatology and Pediatric Hematology/Oncology, Department of Pediatrics, Chang Gung Memorial Hospital, Yunlin 638, Taiwan; 12 School of Dentistry, College of Oral Medicine, Taipei Medical University, Taipei 110, Taiwan

**Keywords:** endocrine disruptors, fine particulate matter, oxidative stress, prostate cancer, reactive oxygen species

## Abstract

Prostate cancer (PCa), a highly prevalent cancer in men worldwide, is projected to rise in the coming years. As emerging data indicate the carcinogenic effects of fine particulate matter (PM2.5) in lung cancer and other site-specific cancers, there is an urgent need to evaluate the relationship between this environmental risk factor and PCa as a potential target for intervention. The present review provides up-to-date evidence about the impact of airborne PM2.5 pollution on the initiation and progression of PCa. Examining the composition and characteristics of PM2.5 reveals its ability to induce toxic effects, inflammatory injuries, and oxidative damages. Additionally, PM2.5 can attach to endocrine-disrupting chemicals implicated in prostatic carcinogenesis. Considering the potential significance of oxidative stress in the risk of the disease, our review underlines the protective strategies, such as antioxidant-based approaches, for individuals exposed to increased PM2.5 levels. Moreover, the findings call for further research to understand the associations and mechanisms linking PM2.5 exposure to PCa risk as well as to suggest appropriate measures by policymakers, scientific researchers, and healthcare professionals in order to address this global health issue.

## 1. Introduction

Prostate cancer (PCa) ranks second by incidence only to lung cancer among men worldwide, leading to significant morbidity and mortality [1]. By 2024, the number of new cases is estimated to double to 2.9 million, whereas the number of deaths is projected to increase by 85% to nearly 700,000 [2]. The development of PCa is linked to complex interactions between inherited germline susceptibility, acquired somatic changes, and environmental variables [3]. The well-established risk factors for the disease include age, ethnicity, and family history, none of which are modifiable [4]. The findings underscore the necessity for further research to better understand the drivers for the upcoming surge in PCa cases and deaths.

Considering the unprecedented urbanization and industrialization globally, air pollution has emerged as one of the greatest scourges in our era. Air pollution, defined as the introduction into the atmosphere of harmful solids, liquids, or gases produced in higher-than-usual concentrations, is linked to many serious health problems, particularly respiratory, cardiovascular, and neurological diseases, cancers, and premature deaths [5,6]. The World Health Organization (WHO) reports that air pollutants of major public health concern include particulate matter (PM), carbon monoxide, ozone, nitrogen dioxide, and sulfur dioxide [7]. PM, a complex mixture of particles with diverse physical and chemical characteristics, is categorized by aerodynamic diameter into coarse (PM10), fine (PM2.5), submicron (PM1) and ultrafine (PM0.1) fractions [8]. A recent review suggests that chronic exposure to PM can compromise every organ in the body, exacerbating existing conditions through toxic, inflammatory, and oxidative mechanisms [9]. In 2013, the specialized cancer agency of the WHO, called the International Agency for Research on Cancer (IARC), classified PM as carcinogenic to humans [10]. Due to the particulate size, PM can easily penetrate deep into the respiratory system and enter the bloodstream, inducing DNA damage, disrupting cellular processes, and promoting the acquisition of biological capabilities required for carcinogenesis at different sites [9]. PM can induce reactive oxygen species (ROS) overproduction that surpasses antioxidant capacity to cause oxidative stress, leading to the damage of mitochondria, endoplasmic reticulum, and DNA; inflammation; the activation of cell death pathways; and even the evasion of immune responses [11,12,13]. As not all PMs are equally toxic, the pathophysiological mechanisms vary between PM species [14]. Given its significant association with a wide range of health issues that led the WHO to designate PM2.5 as a key air particle pollution indicator in 2006, the focus in recent decades has been on this fraction [15]. The WHO estimated that in 2019, up to 99% of the global population was exposed to air pollution levels exceeding its guidelines. This resulted in 4.2 million premature deaths annually worldwide, primarily due to respiratory, cardiovascular, and cancer-related diseases caused by PM2.5 [16]. Recently, the Global Burden of Diseases, Injuries, and Risk Factors Study (GBD) 2021, which provided comprehensive estimates of exposure levels, relative health risks, and the attributable burden of disease for 88 risk factors in 204 countries and territories and 811 subnational locations from 1990 to 2021, further reported that PM air pollution was the leading cause of the global disease burden in 2021, accounting for 80% of total disability-adjusted life years. Ambient PM air pollution also showed the highest increase in the risk-attributable burden among risk factors associated with the leading Level 3 risks [17]. In order to better measure and manage the health risks related to particulate air pollution exposure, PM2.5 air pollution should be investigated as a complex source-driven mixture. Major sources of PM2.5 include vehicle emissions, industrial manufacturing, fuel oil combustion, and biomass burning. PM2.5 constituents mainly consist of black carbon; polycyclic aromatic hydrocarbons (PAHs); heavy metals; and other organic, inorganic, and biological species [18]. It has been found that varying sources and compositions are crucial factors determining particle behavior in the human body. An observational and modelling study, for example, estimated that among the 8.34 million excess deaths per year worldwide due to PM2.5 and ozone air pollution, 5.13 million (61%) were linked to emissions related to fossil fuels [19]. It is well documented that ambient particles from burning fossil fuels have a higher content of harmful metals per unit mass than those from other sources, such as crustal-derived windblown soil [20]. Consistently, a recent review concluded that fossil fuel combustion PM2.5 may have greater potential to cause negative health impacts than other ambient particles [21]. This might be due to the fact that transition metals, such as As, Co, Pb, V, Ni, and Zn, are known to be highly capable of participating in redox processes that result in oxidative stress, contributing to metal toxicity [22]. Moreover, fossil fuel combustion PM2.5 contains varying amounts of sulfur, and the acidic nature of the resulting sulfur compounds can further enhance the bioavailability of constituent transition metals. This pronouncedly raises the capacity of particles to induce oxidative stress and systemic health effects [21]. Aside from heavy metals, the carcinogenic effects of PM2.5 also include the adherence of various organic components such as PAHs, PAH-quinones, and bacterial endotoxins [23]. Despite being reported to be between 20% and 30% on average, the concentrations of organic chemicals may reach as high as 90%. The carcinogenicity of PAHs is mainly attributable to their metabolism and genotoxicity through the formation of reactive electrophilic metabolites to cause DNA adducts, resulting in mutations in both oncogenes and tumor suppressor genes [24]. Therefore, elucidating the impact of ambient PM2.5 pollution may give a deeper understanding of detrimental sources and harmful components as well as provide tools for developing more efficient measures to reduce environmental exposure to PM2.5 pollution, which can help to mitigate health adversity, particularly for individuals at higher risk.

While there is robust evidence linking PM2.5 exposure to lung cancer, it has failed to provide a conclusive association for other cancer sites [25]. The possibility that PM2.5 increases the risk of PCa was proposed with findings of the impact of air pollution on urological diseases [26,27,28,29]. Since PM2.5 is an essential part of air pollutants, the hypothesis that airborne carcinogenesis is involved in PCa development would make sense. In fact, a retrospective population-based study conducted in China from 1982 to 2010 demonstrated significantly positive correlations between industrial waste gas emissions, including PM2.5, and incidence rates of various cancers, among which was PCa (r_s_ = 0.980, *p* < 0.001). However, this research investigated the overall impact of industrial waste gas emissions as a whole mixture rather than a specific pollutant, and PCa was not the cancer of a priori interest [30]. As more epidemiological studies on the association between PM2.5 and PCa have been documented in the past years, it is crucial to recapitulate the evidence. Although the mechanisms are yet to be fully understood, several studies have proposed potential pathways for related cancers. The large surface area and small size of PM2.5 enable the particles to bind to toxic substances and to translocate into the circulatory system, compromising various tissues in the body [14]. It is known that PM2.5 can induce inflammatory responses and produce excess ROS, establishing a synergistical mechanism through which the particles trigger biologically negative effects at the exposed sites [31]. Moreover, PM2.5 can carry endocrine-disrupting chemicals associated with the development of hormone-sensitive cancers [32], such as testicular cancer [33]. The management of PCa continues to evolve rapidly due to substantial progress in understanding the underlying mechanisms. Integration of the knowledge gained so far about PCa carcinogenesis with those pertaining to redox states in the prostatic pathophysiology is demanded [34,35]. Therefore, this review aims to provide up-to-date evidence on the PCa risk that PM2.5 pollution may pose with a focus on epidemiological studies. In addition, our work seeks to elucidate possible mechanisms that may lead from the inhalation of PM2.5 to adverse outcomes, emphasizing the oxidative paradigm. The paper also highlights research gaps that exist in the field and potential directions that research might take in the future.

## 2. PM2.5 Exposure and Prostate Cancer: The Current Epidemiological Evidence

A growing body of epidemiological studies has proven a role for PM2.5 exposure in the initiation and progression of PCa. An association between airborne particulate pollution and PCa was first observed in the United States in 1969, where higher levels of suspended particulate pollution were linked to the increased mortality rates of the disease in older white males [36]. However, this ecological study was unable to control for individual-level risk factors. Moreover, the use of mortality follow-ups may be insufficient to estimate the impact of air pollution on the burden of cancer due to the problem of latency and the possible confounding from mortality of other causes [37]. Therefore, further evidence using cancer incidence rather than mortality has contributed to exploring the health effects associated with PM2.5 exposure. Over the past decade, the relationship between PM2.5 pollution and PCa risk has grown. A large Canadian population-based case-control study (1420 cases and 1424 controls) found a substantial correlation between exposure to ambient PM2.5 over a 20-year period and incident PCa. An interquartile range (IQR) increase in PM2.5 resulted in a 20% to 28% relative increase in PCa risk [38]. Considering a percentage of PCa latent cases, a positive association may indicate higher detection rates as a result of an increased prevalence of PCa screening. In other words, there might be two causes for the positive relationship between PM2.5 exposure and cancer incidence. One is that PM2.5 raises the risk of the disease, and another is that more cancer screening measures do. Consistently, a Chinese study using the time series data of annual incidence and mortality of the ten most common cancers as well as mean PM2.5 concentrations over a 10-year period concluded that PCa was one of the cancers significantly associated with PM2.5 exposure in terms of both incidence and mortality. An analysis of spatiotemporal series data further demonstrated that with every 10 μg/m^3^ increment of annual mean PM2.5 concentrations, the relative risk (RR) of PCa incidence increased by 17% in urban areas but showed no substantial change in rural areas [39].

In order to obtain a more precise exposure–response relationship between PM2.5 and PCa, large-scale prospective cohort studies are required. A nationwide longitudinal cohort study among 87,608 South Korean participants suggested that every 10 μg/m^3^ increase in individual-level PM2.5 concentrations over the previous five years may promote the mortality risk of PCa (hazard ratio (HR) = 1.80, 95% confidence interval (CI): 0.21–15.76) [40]. Similarly, a recent cohort study conducted in the United States also indicated that long-term exposure to PM2.5 increased the risk of PCa within a 10-year period leading up to diagnosis, even at low exposure levels. Importantly, a single-unit decrease in long-term PM2.5 may potentially prevent at least 460 cases per year in the cohort [41].

Recent short- and long-term studies on the relationship between PM2.5 and PCa are summarized in Table 1, the majority of which supports a significantly positive association. While PM2.5 has been the subject of most research, ultrafine particles have not received as much attention. A Canadian population-based case-control study between 2005 and 2009 showed that ambient PM0.1 concentrations were associated with an elevated risk of PCa (odds ratio (OR) = 1.10, 95% CI: 1.01–1.19) [42]. Nonetheless, no long-term studies investigating PM0.1 and PCa can be found to date.

## 3. Fine Particulate Matter, Oxidative Stress, and Prostate Cancer: How Are They Linked?

**Figure 1:**
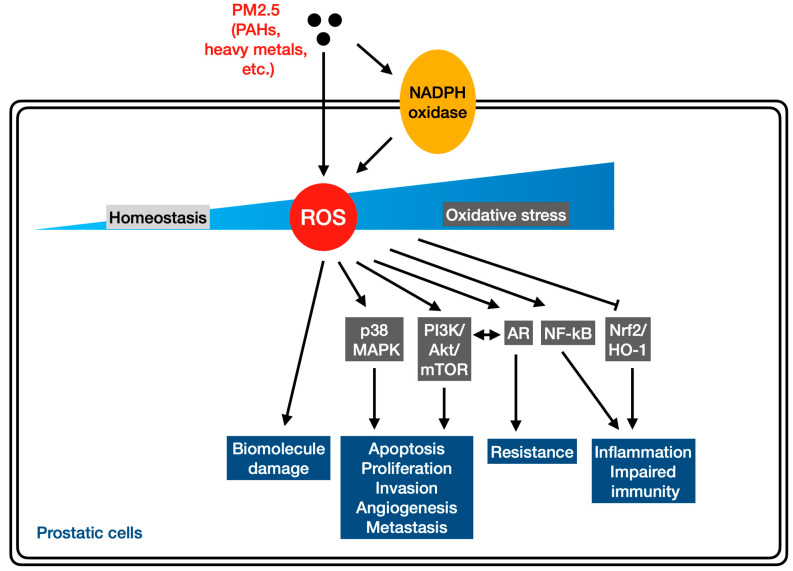
The potential mechanisms through which PM2.5 increases PCa risk with an emphasis on redox biology. PM2.5 can generate ROS both directly and indirectly, which, if not properly counteracted by antioxidant capacity, can lead to oxidative stress. As second messengers, ROS can dysregulate varying redox-sensitive signaling transduction pathways, including the mitogen-activated protein kinases (MAPKs) and phosphoinositide 3-kinase/protein kinase B (PI3K/Akt), that are involved in multistage carcinogenesis. Redox states also play an important role in immunity and T-cell activity, in which ROS levels determine immune responses. ROS overproduction may enhance the release of proinflammatory cytokines orchestrated and regulated by many redox-sensitive transcription factors, such as the NF-κB and Nrf-2. In addition, some genetic alterations during PCa progression may contribute to the activity of the androgen receptor (AR), whose regulation exhibits a reciprocal negative feedback mechanism with PI3K/Akt signaling. Interestingly, PM2.5 may have an endocrine-disrupting potential, presenting another exposure source to endocrine disruptors implicated in prostatic carcinogenesis.

Since its introduction in 1985 [52], oxidative stress is widely defined as “an imbalance between oxidants and antioxidants in favor of the oxidants, leading to a disruption of redox signaling and control and/or molecular damage”. Chemically reactive molecules of low molecular mass, also known as reactive species, have been extensively studied in their role in redox regulation, among which ROS is one of the key players [53]. It is known that excessive ROS production, or a deficient antioxidant defense system, or both can push the cell to undergo oxidative stress, triggering various cellular processes linked to the initiation and progression of many cancers, including PCa [54]. Apart from damaging DNA, proteins, and lipids, oxidative stress can modulate gene expression, induce epigenetic alterations, and even modify posttranslational indicators, resulting in signaling disruption, cellular dysfunction, and malignant predisposition [55]. Although the association between PM2.5 and PCa may imply a causal relationship, the mechanisms through which the particles may contribute to prostatic carcinogenesis have yet to be fully understood. A growing body of evidence has demonstrated the significance of ROS in the development of PCa. Higher levels of intracellular ROS, including superoxide and hydrogen peroxide, were found in human PCa cell lines rather than in prostate epithelial cell lines. In addition, the inhibition of ROS production from the NAPDH oxidase system by using a pharmacological inhibitor dramatically attenuated the cell migration ability, anchorage-independent colony formation, and cell proliferation of PCa cells, suggesting an essential role of ROS in malignant cell behaviors [56]. Consistently, increased levels of hydrogen peroxide and NADPH oxidase 1 expression were observed in human PCa tissue samples [57]. Moreover, oxidative stress has been well recognized as one of the primary mechanisms underlying PM2.5 health effects, including carcinogenicity [11,18]. It is known that PM2.5 can reach the systemic circulation for further translocation to other parts in the body upon inhalation through the airway, resulting in adverse effects at both cellular and molecular levels [14]. Although there is currently no evidence that PM2.5 is directly detected in the male prostate, recent studies have shown that the particles can accumulate in the reproductive organs through the blood–testis barrier, –placental barrier, –epithelial barrier, and other barriers protecting reproductive tissues [58]. Therefore, PM2.5-induced oxidative stress may be a critical paradigm. Figure 1 depicts the possible mechanisms through which the exposure to PM2.5 pollution can increase the risk of PCa, focusing on redox biology, as discussed in more detail below.

It is known that PM2.5 can directly generate ROS, such as hydroxyl radicals, through the redox cycling of environmentally persistent free radicals presenting in the particles [59]. Moreover, PM2.5 contains a variety of carcinogens and toxic components, such as heavy metals and PAHs, which may enable the induction and progression of PCa [60,61]. Toxicological research has investigated the inflammatory and oxidative effects of such substances in the context of prostate health. An animal study found that cadmium (Cd), a trace metal content in PM2.5, can cause reproductive toxicity and induce prostatic deficiency. The mechanisms included the induction of prostatic inflammation, oxidative stress, and an epithelial–mesenchymal transition; activation of the TGF-β1/Smad pathway; a reduction in the Bcl-2/Bax ratio; and inhibition of the Nrf-2/HO-1 pathway [62]. Similarly, chronic cigarette smoke exposure was reported to cause prostate deficits by inducing local inflammation, oxidative stress, and epithelial–mesenchymal transitions [63]. In addition, PAHs are known as redox-active species presenting in PM2.5, which lead to ROS production through the quinone redox cycle [55,64]. An in vitro study using prostate-derived cell lines from localized adenocarcinoma and bone metastasis as well as non-neoplastic prostate epithelium cells reported that PAHs can stimulate cell growth, particularly in localized cancer cells, and can increase VEGF and HIF expression as well as ROS production. In addition, the data demonstrated that toxic concentrations of PAHs were associated with GSH depletion, indicative of oxidative stress [65]. These findings suggest that PAH exposure may contribute to PCa progression, in part due to ROS overproduction. Therefore, cumulative biological changes triggered by long-term exposure to PM2.5 and its active constituents may contribute to a multistage prostatic carcinogenesis process. Additional studies, however, should be conducted to determine PM2.5 concentrations that reach the carcinogenic effects. Considering currently available measures for evaluating the oxidative potential of particulate air pollution, future studies should also investigate whether particle oxidative potential measurements are more markedly related to cancer risk than traditional mass- or number-based exposure metrics [66].

Since ROS act as second messengers in various signal transduction pathways, exposure to PM2.5 can dysregulate multiple redox-sensitive signaling pathways associated with carcinogenesis, among which the mitogen-activated protein kinases (MAPKs) and phosphoinositide 3-kinase/protein kinase B pathways stand out [11]. The MAPK pathway, consisting of ERK1/2, JNK, and p38 MAPK, plays a role in cell proliferation, differentiation, and apoptosis. Several studies have shown a potential connection between p38 MAPK and PCa [67,68]. In addition, the PI3K/AKT/mammalian target of the rapamycin (mTOR) pathway is considered a pivotal intracellular signaling pathway, whose hyperactivity is also linked to carcinogenesis. The de-regulation of the PI3K/AKT/mTOR pathway was found in 42% of localized and 100% of advanced PCa cases, suggesting that any factor that may disrupt this pathway is predictive of disease progression [69].

Recently, the alarming health impacts induced by endocrine disruptors (EDs) have drawn attention from all around the world. Although ingestion is known as the primary exposure route, inhalation has been proposed as an important route [70]. A recent systematic review about the in vitro endocrine activity of ambient PM has reported that the particles can induce estrogenic, antiestrogenic, androgenic, and antiandrogenic effects. The findings suggest that PM may have an endocrine-disrupting potential, posing an additional exposure source to EDs [71]. Although it is difficult to estimate the amount that inhalation can contribute to the total burden of EDs, the endocrine activity of PM may worsen health issues. Bioassay-based assessments may be a useful tool to measure the health risk caused by airborne EDs. In fact, PAHs can promote the growth of the breast cancer cell line MCF-7, which is mediated by the estrogen receptor [72]. Such receptors may also be expressed in benign and malignant prostate epithelial cells [73]. Furthermore, PAHs may function as agonists to interact with the androgen receptor (AR) [74]. It is well recognized that the majority of PCa cases are dependent on androgen stimulation mediated by AR for cell growth and survival. Additionally, androgens may enhance ROS levels [75]. Interestingly, AR and PI3K/Akt signaling regulation show a reciprocal negative feedback mechanism, in which the inhibition of one inactivates the other, resulting in cancer cell survival and progression [76]. Thus, bypassing the AR pathway associated with androgen independence may be employed as an alternative for PCa survival [77]. However, other genetic alterations during PCa progression may contribute to AR activity, which accounts for high androgen-receptor sensitivity in response to androgens, antiandrogens, or nonandrogenic hormones, providing a selective growth advantage to PCa cells [78].

There is increasing evidence indicating that PM2.5 exposure can impair the immune system, contributing to the development of cancers [79]. Although PCa is not classified as an immunologically responsive tumor, the interaction between prostatic epithelial cells with both immune and non-immune cells that make up the tumor microenvironment (TME) still plays an important role in the disease progression and overall resistance to treatment [80]. The mechanisms through which PCa cells can evade the immune system, maintain the “cold” TME, and mediate immunosuppressions are yet to be elucidated. So far, the literature has reported that redox states have important roles in immunity and T-cell activity, whereby the ROS levels may determine immune responses [81,82]. A mild increase in ROS levels in the immune system may facilitate normal immune function, whereas moderate ROS levels can act as the biochemical mediators in immunity involved in multiple cellular functions and signaling pathways. In contrast, high ROS levels may result in a rise in the release of proinflammatory cytokines orchestrated and regulated by various redox-sensitive signaling pathways [83,84]. The NF-κB family of transcription factors that plays a role in inflammation and immunity can be regulated by ROS [85]. It has been reported that PCa cells may exhibit constitutive NF-κB activity due to the increased activity of the IκB kinase complex, which is inversely associated with AR activity [86,87]. In addition, the function of immune cells is regulated under redox control through the activity of Nrf-2 and cellular antioxidants [88,89]. An in vivo study demonstrated that the progression of prostate tumors was associated with methylation silencing of the Nrf2 promoter as well as decreased transcription of Nrf2 and Nrf2 target genes [90]. The incentive for gaining a better understanding of the TME and immune resistance mechanisms is necessary to further explore the adverse effects of PM2.5-induced oxidative stress.

## 4. Practical Implications: Mitigation of PM2.5 Oxidative Effects on Prostate Cancer

The fact that oxidative stress is higher in PCa patients than healthy men suggests that antioxidants may play a crucial role in preventing disease progression [91,92]. Antioxidants are substances able to counteract the free radical generation and oxidation process, which can be classified by their source into endogenous sources, such as enzymes, and exogenous sources, such as beta-carotene; lycopene; and vitamins A, C, and E (tocopherols) [93]. An in vitro study indicated that ROS production rather than accumulation affected PCa phenotypic behavior, implying that antioxidant-based approaches may not be beneficial, since antioxidants can only neutralize the accumulated ROS within the cells [56]. A number of observational studies have examined the effects of dietary antioxidants on the initiation and progression of PCa [94,95,96,97,98,99]. Some clinical trials concluded that supplemental dietary antioxidants had no substantial impact on the overall risk for PCa [96,97,98], whereas smokers who took 50 mg of vitamin E daily had a statistically significant 32% lower PCa incidence and 41% lower PCa mortality than those who received a placebo [95]. A recent review reports that most studies have focused on carotenoids, particularly beta-carotene and lycopene, vitamins E and C, phenolic dietary sources such as coffee and tea, and flavonoids. Overall, many of these studies were elusive and equivocal about the actual benefits, since different antioxidants show varying effects on PCa risk [100]. Interestingly, consuming a diet rich in fish, legumes, fresh fruits, and vegetables, along with vitamin D3 supplements, is recommended in areas with high air pollution levels [101]. Although existing data do not provide sufficient support for a population-wide implementation of antioxidant supplementation against PCa, intervention approaches aimed at reducing ROS production still might offer an effective strategy for the prevention of PCa from PM2.5 exposure. Therefore, subjects exposed to high concentrations of ambient PM2.5 should consider mitigating oxidative stress by increasing antioxidant intake through their diet and/or supplements. Antioxidant administration via inhalation is being studied as a promising strategy to protect against oxidative damage caused by air pollution [102].

Androgen deprivation therapy (ADT), which involves either surgical or pharmacological castration to reduce the production and/or action of androgens, remains the first-line treatment for metastatic PCa. While the AR signaling axis is considered to be primarily responsible for castration-resistant PCa, another avenue of research has focused on oxidative stress [103]. A recent study shows that castration can result in dramatic increases in the activity of ROS-generating NADPH oxidases [104]. As increased NADPH oxidase-driven ROS generation can lead to the generation of a malignant phenotype in PCa by modulating various signaling cascades [105], this emerging candidate may prove to be an effective target for therapeutic intervention.

On a broader scale, initiatives to avoid increased exposure to PM2.5 may include enacting and enforcing air pollution regulations, switching to renewable energy sources and encouraging public transit. The utility of cancer screening programs and routine examinations should not be overlooked for individuals residing in areas with high concentrations of ambient PM2.5. Moreover, in our rapidly urbanizing world, green space has shown potentially beneficial effects on human health through a reduction in noise, heat, and air pollution; motivation for physical activity; and improvements in psychophysiological health [106]. A large Taiwanese population-based cohort study on the association between greenness and cancer incidence demonstrated the protective effect of greenness against incident PCa [47]. In line with these findings, a previous Canadian population-based case-control study also suggested that men living in greener areas had a lower risk of PCa [107]. Although the exact impact of green space on PCa remains vague, it is thought that the burden of genetic and epigenetic responses leading to carcinogenesis can be attenuated as less inhaled toxicants reach the tissues.

## 5. Challenges and Future Research

Although current epidemiological studies have provided evidence on the potential relationship between PM2.5 exposure and the PCa course, several gaps and directions for future research exist. Addressing these challenges would help to gain a deeper understanding of the PCa risk associated with PM2.5 pollution and to develop effective strategies to protect prostate health.

First, it is difficult to demonstrate causality between the exposure to PM2.5 and the development of PCa because of the long latency. Moreover, most of the epidemiological evidence to support this association derives from observational studies, in which the results might be biased by many confounding factors. There were probably measurement errors across studies due to the lack of personal-level exposure information. Compared to ecological and case-control studies, longitudinal cohorts would yield the most reliable findings due to the prospective collection of individual-level data. The existing proofs also did not consider the location of participants (outdoors, at home, or at work) and their movement/migration throughout the study period. As previously stated, varying PM2.5 sources and components are significant determinants for the particle impact on our health. To pave the way for a better understanding of the relationship between PM2.5 exposure and PCa risk, further research that captures individual-level exposure, long-term follow-up, different groups of susceptible populations, varying source-specific PM2.5 effects, and covariate variables is warranted. This may provide tools for developing more effective ways to attenuate the health effects related to PM2.5 exposure in humans, particularly for those who are more vulnerable.

Second, the discrepancy in the magnitude of PM2.5 effects can be due to varying exposure ranges. Nearly half of the studies included in the present review were conducted in regions with relatively low PM2.5 levels, including Europe and North America. Additional studies should be prioritized in developing countries, such as Asian and South American countries, where PM2.5 concentrations are higher.

Third, the global prevalence of PCa differs among various geographical regions and ethnic groups. Although black men have the highest incidence rates of PCa in the world [1,2], little is known about the impact of PM2.5 pollution on PCa risk in African nations. More studies should consider the genetic background, socioeconomic status, and climate to determine possible responses leading to geographical and racial changes in PCa rates associated with PM2.5 exposure.

Last but not least, the single-pollutant model might not be able to reveal potential interactions between air pollutants. Future studies should implement mixture models to investigate the concurrent exposure to multiple air pollutants and the time–microenvironment–activity paradigm. Above all, the underlying mechanisms and potential factors mediating PCa risk require further research to tailor intervention strategies to the specific context of PM2.5 pollution.

## 6. Conclusions

The ubiquity of airborne PM2.5 pollution poses a serious public health concern worldwide, since it has numerous adverse effects on human health, including a potentially increased risk of PCa. As research continues, it is imperative to implement additional studies from basic science to population-level investigations to uncover the intricate mechanisms linking PM2.5 exposure to PCa development. The collaboration between policymakers, scientific communities, and healthcare professionals is crucial to formulating comprehensive strategies that protect prostate health from the impact of PM2.5 pollution.

## Figures and Tables

**Table 1:** Summary of recent epidemiological research on the association between PM2.5 and PCa.

Study					PM2.5 Exposure (µg/m^3^)		Results		Ref
Authors (Year)	Location	Design	Time Period	Number of Participants	Average (S.D)	Range	Outcome	Results	
Felici et al. (2024)	UK	Case-control	2006–2010	Cases: 12,838 Controls: 141,596	N/A	N/A	Incidence	No significant association: OR = 0.982, 95% CI = 0.962–1.002, *p* = 0.072 per 1 μg/m^3^ increase	[43]
Kayyal-Tarabeia et al. (2024)	N/A	Ecological	2007–2015	30,979	N/A	N/A	Incidence	HR per an IQR increase in PM2.5 (2.11 µg/m^3^): 1.41 (95% CI: 1.31–1.52)	[44]
Thomas et al. (2024)	N/A	Cohort	1994–2017	43,184	N/A	N/A	Incidence	No significant association: HR = 1.02, 95% CI = 0.95–1.08 per every 5 μg/m^3^ increase	[45]
Fan et al. (2023)	China	Ecological	2015–2020	N/A	60.3	40.2–81.6	Mortality	RR for the association with a 1 μg/m^3^ increase in long-term exposure to PM2.5: 1.089 (95% CI: 1.034–1.148)	[46]
Wei et al. (2023)	US	Cohort	2000–2016	2,161,156	9.8	0.0–30.9	Incidence	Absolute increase in the risk of cancer diagnosis per unit increase in PM2.5: 0.0112% (95% CI: 0.0094%–0.0131%)	[41]
Huang et al. (2022)	Taiwan	Cohort	2000–2015	407,415	20.89	N/A	Incidence	HR per every 10 μg/m^3^ increase in the 2-year-average PM2.5 concentration: 0.96 (95% CI: 0.84–1.08)	[47]
Shin et al. (2022)	Korea	Cohort	2007–2015	87,608	N/A	N/A	Mortality	HR per every 10 μg/m^3^ increase in individual-level PM2.5 concentrations for the previous 5 years: 1.80 (95% CI: 0.21–15.76)	[40]
Youogo et al. (2022)	Canada	Case-control	1994–1997	Cases: 1420 Controls: 1424	N/A	N/A	Incidence	An IQR increase in PM2.5 (3.56 µg/m^3^ for satellite and 4.48 µg/m^3^ for scaled satellite observations) yielded ORs of 1.28 (95% CI: 1.07–1.52) and 1.20 (95% CI: 1.03–1.40), respectively	[38]
Yu et al. (2022)	Brazil	Ecological	2010–2018	127,449	7.63	3.37–21.02	Mortality	RR for the association with a 10 μg/m^3^ increase in 3-year-average PM2.5: 1.18 (95% CI: 1.05–1.32)	[48]
Yu et al. (2022)	Brazil	Ecological	2010–2016	96,501	2.38	0.60–12.49	Mortality	RR for the association with a 1 μg/m^3^ increase in 2-year-average wildfire-related PM2.5: 1.03 (95% CI: 1.01–1.06)	[49]
Coleman et al. (2020)	US	Cohort	1987–2014	635,539	N/A	N/A	Mortality	HR per every 10 µg/m^3^ increase in PM2.5: 0.91 (95% CI: 0.68–1.22)	[50]
Coleman et al. (2020)	US	Ecological	1992–2016	1,151,454	11.50	N/A	Incidence	Incidence rate ratio for the association with a 10 μg/m^3^ increase in PM2.5: 0.96 (95% CI: 0.87–1.06)	[37]
Wang et al. (2019)	China	Ecological	2006–2009	136	N/A	N/A	Incidence Mortality	RR of PCa incidence in urban area increased by 17% with a 10 μg/m^3^ increase in annual mean PM2.5 concentration	[39]
Turner et al. (2017)	US	Cohort	1982–2004	623,048	12.6	1.4–27.9	Mortality	HR per every 4.4 μg/m^3^ increase in PM2.5: 0.96 (95% CI: 0.86–1.06)	[51]

CI: confidence interval; HR: hazard ratio; IQR: interquartile range; OR: odds ratio; RR: relative risk; S.D.: standard deviation. N/A: Not Applicable.

## Data Availability

The manuscript contains all relevant data.
